# THE SENESCENCE-ASSOCIATED SECRETOME AS A BIOMARKER OF AGING

**DOI:** 10.1093/geroni/igae098.0985

**Published:** 2024-12-31

**Authors:** Nathan LeBrasseur

**Affiliations:** Mayo Clinic, Rochester, Minnesota, United States

## Abstract

Senescent cells accumulate in nearly all organs with advancing age. A defining characteristic of senescent cells is a robust and heterogenous senescence-associated secretory phenotype (SASP). The SASP influences tissue health and function locally and systemically, hence, circulating components of the SASP may be informative of senescent cell burden. We established a candidate panel of senescence biomarkers based on common cytokines, chemokines, matrix remodeling proteins, growth factors and other proteins components of the SASP. In this seminar, the associations between senescence biomarkers and clinical manifestations of biological age, including frailty, functional limitations, and chronic disease, in humans will be presented. Moreover, the responsiveness of senescence biomarkers to interventions that counter the biology of aging, including exercise and caloric restriction, will be highlighted. Finally, the strengths and limitations of this approach and new directions will be discussed.

